# Keeping Intergenerational Programming Alive During the Pandemic Through Collaboration and Technology

**DOI:** 10.1093/geroni/igab046.1555

**Published:** 2021-12-17

**Authors:** Erica Estus, Catherine Taylor, Skye Leedahl

**Affiliations:** 1 University of Rhode Island, Kingston, Rhode Island, United States; 2 Age-Friendly Rhode Island, Providence, Rhode Island, United States

## Abstract

The University of Rhode Island Cyber-Seniors’ in-person intergenerational programming was quickly shut down during the Spring 2020 semester due to the pandemic. Since then, we have worked diligently and collaboratively with partners to offer creative intergenerational options for university students and older adults living in the community. We partnered with Age-Friendly RI and the Census Outreach to provide phone-based wellness checks to 11,500 older adults, and this evolved into a statewide weekly call with partners (n=34 calls) focused on reducing social isolation for older adults. Our students moved to offering phone or Zoom-based appointments with 21 community organizations across Rhode Island and became mentors for the new Cyber-Seniors ® organization digital offerings (n=90 students). In this presentation, we will share our experiences with the pivot from in-person to mostly technology-based interactions. We will discuss challenges and lessons learned, some of which will be retained regardless of the pandemic situation.

